# Should I stay or should I go? Leveraging data‐driven approaches to explore the effect of various disaster policies on postearthquake household relocation decision‐making

**DOI:** 10.1111/risa.70007

**Published:** 2025-03-11

**Authors:** Chenbo Wang, Gemma Cremen, Carmine Galasso

**Affiliations:** ^1^ Department of Civil, Environmental and Geomatic Engineering University College London London UK

**Keywords:** data‐driven approaches, disaster risk reduction policies, postearthquake household relocation, pro‐poor, risk‐sensitive urban development

## Abstract

Devastating earthquakes can cause affected households to relocate. Postearthquake relocation disrupts impacted households' social ties and, in some instances, their access to affordable services. Simulation‐based approaches that model postearthquake relocation decision‐making can be valuable tools for supporting the development of related disaster risk reduction (DRR) policies. Yet, existing versions of these models focus particularly on housing‐related factors, which are not the sole driver of postearthquake relocation. We integrate data‐driven approaches and local data to account for postearthquake household relocation decision‐making within an existing simulation‐based framework for policy‐related risk‐sensitive decision support on future urban development. We use household survey data related to the 2015 Gorkha earthquakes in Nepal to develop a random forest model that estimates the postearthquake relocation inclination of disaster‐affected households. The developed model holistically captures various context‐specific factors important to postearthquake household relocation decision‐making. We leverage the framework to quantitatively assess the effectiveness of various DRR policies in reducing positive postearthquake relocation inclination, with an explicit focus on low‐income households. We demonstrate it using “Tomorrowville,” a hypothetical expanding urban extent that reflects important social and physical characteristics of Kathmandu, Nepal. Our analyses suggest that the provision of livelihood assistance funds is more successful when it comes to mitigating positive postearthquake relocation inclination than hard policies focused on strengthening buildings (at least in the context of the examined case study). They also suggest viable pro‐poor pathways for mitigating disaster relocation impacts without the need to create potentially politically sensitive income‐based restrictions on policy remits.

## 1 INTRODUCTION

Moderate‐to‐large earthquake events can adversely impact vulnerable urban environments, often resulting in significant disruptions to social and economic activities. Affected households may subsequently decide to relocate (e.g., Binder et al., 2015), as observed following major past seismic events, for example, the moment magnitude (M) 6.9 Loma Prieta, California, USA, earthquake (Schwab et al., 1998), the M8.0 Wenchuan, China, earthquake (Ge et al., 2010), and the M7.8 and M7.3 Gorkha, Nepal, earthquakes (He et al., 2018; Wilson et al., 2016). Postdisaster relocation often causes emotional instability, distress, depression, trauma, and other psychological effects among those who relocate (Bier, 2017; Kılıç et al., 2006; Makwana, 2019). It also has long‐lasting impacts on the social ties of relocated households and, in some instances, can deprive them of access to affordable housing, employment, healthcare, and education for years and even decades after relocation (Badri et al., 2006). Furthermore, earthquake disasters have historically led to disproportionate relocations of socioeconomically vulnerable households, for example, female‐headed households, the elderly, racial and ethnic minorities, and the urban poor (Bier, 2017; Hunter, 2005; Morrow‐Jones & Morrow‐Jones, 1991; Myers et al., 2008). Inequities are further exacerbated by the additional relocation‐induced implications and vulnerabilities that result. Therefore, stakeholders (e.g., urban planners, recovery planners, and emergency response authorities) must devise strategic disaster risk reduction (DRR) policies for mitigating positive postearthquake relocation decision‐making.

Simulation‐based modeling approaches that capture postearthquake relocation decision‐making are useful tools that complement empirical studies in supporting the design of such DRR policies (e.g., Costa & Baker, 2022; Moradi & Nejat, 2020). For instance, Miles and Chang (2011) developed the *ResilUS* computational model based on fragility models and Markov chains to simulate community‐based postdisaster housing recovery. *ResilUS* models households' decisions to leave or stay accounting for factors predominantly related to housing reconstruction (e.g., the debt incurred from housing repairs and the availability of temporary housing until housing repairs are finished). Nejat and Damnjanovic (2012) proposed an agent‐based model using game theory to predict homeowners' decision‐making (i.e., stay and repair or sell and leave) based on their neighborhood's average reconstruction value and the predicted future reconstruction value. Moradi and Nejat (2020) presented the *RecovUS* spatial agent‐based model to simulate households' decision‐making (e.g., stay and repair, stay and wait for repairs, and sell and leave) accounting for various factors, for example, income, race, education, residential building damage, financial assistance, restoration of community assets and infrastructure, and neighbors' repair progress. Households are assumed to stay and repair if they have abundant financial resources to cover repair costs. Costa, Haukass, et al. (2022) proposed an agent‐based model for assessing temporary displacement and permanent relocation decision‐making of households that centers on aspects related to the immediate built environment, for example, availability of water and electricity, neighborhood conditions, housing repair progress, neighbors' decisions, and socioeconomic factors. Costa, Wang, et al. (2022a) further integrated place attachment (classified as “low” if both neighborhood and housing satisfaction are below a certain threshold) into the assessment of households' decisions to stay and repair or relocate. Low‐income households, renters, and those occupying old buildings were identified as most likely to have low place attachment and, therefore, most prone to relocation (at least within the context of the San Francisco, California, USA, case study considered).

Thus, most existing simulation‐based models for postearthquake household relocation decision‐making focus mainly on housing‐related factors, including but not limited to the duration and costs of housing repairs, whether or not the household can afford these costs, and the availability and affordability of temporary housing while their home is under repair. This means that the models either neglect or do not give adequate attention to alternative factors that can motivate or discourage households from relocating, for example, earthquake‐induced livelihood impact. Many of these models have not been validated with empirical data or are only partially calibrated using highly aggregated relocation patterns observed after past earthquake events (Costa, Haukass, et al., 2022; Costa, Wang, et al., 2022a; Miles & Chang, 2011; Nejat & Damnjanovic, 2012). Therefore, further research is needed to improve the understanding and modeling of postearthquake household relocation decision‐making.

We aim to address this challenge using a data‐driven modeling approach that integrates a holistic range of context‐specific factors to estimate postearthquake household relocation decision‐making. Data‐driven approaches (e.g., logistic regression, random forest, and regression kriging) have been previously used in the literature to develop models for assessing (Costa, Wang, et al., 2022b; Loos et al., 2023; Nejat & Ghosh, 2016; Nejat et al., 2020; Rosenheim et al., 2021) or identifying factors related to (Binder et al., 2015; Myers et al., 2008; Zhang & Peacock, 2009) households' postdisaster behaviors as well as to track business recovery (Costa & Baker, 2021) and to estimate postearthquake damage (Loos et al., 2020). However, these studies either (1) did not explicitly focus on relocation; or (2) considered data at more aggregated resolution (i.e., neighborhood‐ or county‐level) than individual households; (3) predominantly centered on the aftermath of wind‐hazard events (e.g., hurricane) rather than (potentially more devastating) earthquake disasters; and (4) developed models specifically targeted at high‐income locations that may not reflect Global South contexts. The proposed data‐driven model, which overcomes these limitations, is integrated into an existing framework for policy‐related risk‐sensitive decision support on future urban development (Wang et al., 2023). The resulting enhanced framework can then be used to quantify the effectiveness of various DRR policies in mitigating households' decisions to relocate after an earthquake, with an explicit focus on the extent to which low‐income households are impacted. We use Nepali household survey data related to the 2015 M7.8 and M7.3 Gorkha earthquakes to develop the required data‐driven model. We leverage the model to demonstrate the enhanced framework using the “Tomorrowville” virtual urban testbed, which closely reflects important physical and social characteristics of Kathmandu.

We structure this paper as follows. We introduce the enhanced simulation‐based framework in Section 2. We describe the data‐driven model developed for the case study in Section 3, present the case‐study application in Section 4, and provide results in Section 5. Finally, we offer some concluding remarks in Section 6.

## 2 PROPOSED SIMULATION‐BASED FRAMEWORK

**FIGURE 1:**
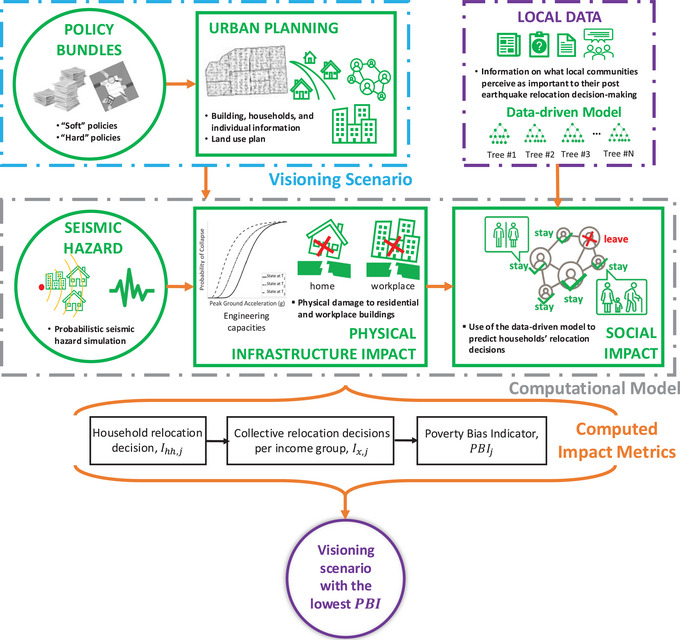
Simulation‐based framework for quantitatively assessing the effectiveness of disaster risk reduction (DRR) policies in mitigating household decisions to relocate after an earthquake.

We advance the existing framework proposed in Wang et al. (2023) to explicitly account for postearthquake household relocation decision‐making, as shown in Figure 1. The original Wang et al. (2023) framework leveraged the Tomorrow's Cities Decision Support Environment (Cremen et al., 2023) and facilitated the development of compulsory household‐level financial soft policies (e.g., insurance, tax relief) for reducing disaster risk in expanding urban areas. The enhanced framework encompasses seven modules: (1) **Policy Bundles**; (2) **Urban Planning**; (3) **Local Data**; (4) **Seismic Hazard**; (5) **Physical Infrastructure Impact**; (6) **Social Impact**; and (7) **Computed Impact Metrics**. (1), (2), (4), (5), (6), and (7) are modified versions of modules within the original framework. The characterisation of postearthquake household relocation decision‐making is facilitated by the new **Local Data** module and its accompanying **Data‐driven Model**.

Stakeholders first design DRR policies (in the **Policy Bundles** module) and apply these policies to a (conditional) urban plan associated with a specific time instance (in the **Urban Planning** module), both of which collectively produce a **Visioning Scenario**. A **Visioning Scenario** represents an urban system at a snapshot in time. While this could be the current version of the urban system, it is intended for the framework to be used in a forward‐looking manner. The information stored in the **Visioning Scenario** and **Local Data** informs the calculations of modules (4) to (6), which collectively comprise the **Computational Model**. Modules (4) to (6) produce seismic hazard calculations, physical infrastructure impacts, and social impacts, respectively. The **Local Data** module provides relevant context‐specific information on household relocation decision‐making. This information informs the development of a **Data‐driven Model**, which is used within the **Social Impact** module to estimate whether households decide to relocate or stay. These estimations are then translated into a Poverty Bias Indicator (PBI), which measures the extent to which low‐income households disproportionately decide in favor of relocation. Each iteration of the framework produces an assessment of impacts for one specific **Visioning Scenario**. The optimal **Visioning Scenario** is the one that produces the lowest PBI. We use Monte Carlo sampling to capture uncertainties within modules (4) to (6), in line with Cremen et al. (2022). Most modules introduced in Wang et al. (2023) are only briefly discussed. Described in detail are the newly introduced **Local Data** module and the accompanying **Data‐driven Model**, the enriched **Social Impact** module, and the **Computed Impact Metrics** that depend on the **Social Impact** module.

### 2.1 Brief description of some modules in the original framework

The **Urban Planning** module contains an urban plan that provides detailed information on land use, buildings, households, and individuals associated with a specific urban area at a prescribed time (possibly in the future, Menteşe et al., 2023). Within the context of the proposed enhanced framework, the **Policy Bundles** module encompasses one or more DRR policies that broadly aim at mitigating decisions to relocate after an earthquake. These policies could be “soft” (e.g., postearthquake livelihood assistance funds) as well as “hard” (e.g., upgrading of existing infrastructure facilities to higher building codes). For this particular study, the **Seismic Hazard** module stores the seismic source and rupture features of a specific earthquake event (scenario). It estimates the resulting ground‐motion intensity measures (IMs) at the locations of exposed assets (e.g., buildings), that is, ground‐motion fields (GMFs). The **Physical Infrastructure Impact** module uses the GMF outputs from the **Seismic Hazard** module in combination with fragility models to estimate physical damage to buildings. This damage is represented as a discrete damage state (DS). The reader is referred to Sections 2.1 to 2.4 in Wang et al. (2023) for more details on these modules.

### 2.2 Local Data

We integrate **Local Data** to allow for context‐specific people‐centered characterization of postearthquake household relocation decision‐making. The **Local Data** module includes information (e.g., household relocation survey data, government reports, and social media information, which may have been gathered from previous community‐driven research/interaction) on how various locally‐relevant factors (e.g., socioeconomic features) relate to household relocation decisions. This knowledge is then used to calibrate a predictive **Data‐driven Model** for relocation decision‐making.

#### 2.2.1 Data‐driven Model

The **Data‐driven Model** estimates postearthquake household relocation decisions. The postearthquake relocation decision for the hhth household in the jth Monte Carlo sample, $I_{hh,j}$, is binary. $I_{hh,j}=1$ means the hhth household decides to relocate and $I_{hh,j}=0$ indicates otherwise. It is developed by applying statistical learning methods (e.g., logistic regression, random forests) to the **Local Data**. The **Data‐driven Model** is therefore inherently context‐specific, enabling a more accurate characterization of postearthquake household relocation decision‐making compared to generic, heuristic models.

### 2.3 Social Impact

The **Social Impact** module uses outputs from the **Physical Infrastructure Impact** module and leverages the **Data‐driven Model** to capture the postearthquake relocation decision‐making of each household ($I_{hh,j}$), considering the policies that feature within the **Policy Bundles** module. This module further computes collective relocation decisions made by households across different income groups ($I_{x,j}$). $I_{x,j}$ for the jth Monte Carlo sample is computed as

(1) $$I_{x,j}=\frac{\sum I_{hh,j,x}}{n_{x}},$$

where x refers to low‐ (low), middle‐ (mid), high‐ (high), or all‐ (all) income groups, $I_{hh,j,x}$ is the hhth household relocation decision associated with income group x, and $n_{x}$ is the total number of households within income group x.

### 2.4 Computed Impact Metrics

The **Computed Impact Metrics** module translates the $I_{x,j}$ outputs from the **Social Impact** module into a single‐valued $PBI_{j}$, which measures the extent to which low‐income households disproportionately decide in favor of relocation. That is

(2) $$PBI_{j}=\frac{I_{low,j}}{I_{all,j}}-1.$$

A negative value of $PBI_{j}$ implies that the policies within the **Policy Bundles** module (and thus the associated **Visioning Scenario**) are pro‐poor, that is, the specific earthquake scenario considered does not result in a disproportionate number of decisions to relocate among low‐income households. See Section 2.6 in Wang et al. (2023) for more details on PBI.

## 3 DEVELOPMENT OF A DATA‐DRIVEN MODEL

We demonstrate the **Local Data** module by developing a **Data‐driven Model** to characterize the relocation inclination of Nepali households after the 2015 M7.8 and M7.3 Gorkha earthquakes. The model estimates relocation inclination (i.e., willingness to relocate) as a proxy for a more definitive relocation decision, due to the constraints of the data set adopted as **Local Data** (see Section 3.1). This data set comprises household‐level survey data related to the 2015 Gorkha earthquakes, which were collected in the 11 districts most affected by these events outside of the Kathmandu Valley. We note the model is developed specifically for households in Kathmandu, and should not be used outside this remit without further context‐specific investigations (to gather local data).

### 3.1 Description of Local Data

**FIGURE 2:**
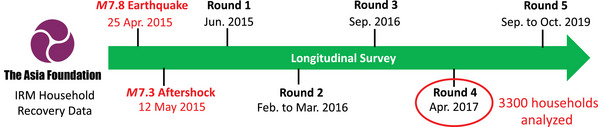
The timeline of the Independent Impacts and Recovery Monitoring (IRM) project, a 5‐year longitudinal study conducted by The Asia Foundation. IRM was designed to systematically monitor disaster‐induced social impacts, recovery patterns, and disaster‐affected households' evolving needs after two devastating earthquakes struck Nepal in April (M7.8) and May (M7.8) 2015. The fourth‐round data, containing 3300 complete responses, are used in this case study.

The **Local Data** data set is derived from the results of the Independent Impacts and Recovery Monitoring (IRM) project, a longitudinal study conducted by The Asia Foundation to systematically monitor disaster‐induced social impacts, recovery patterns, and disaster‐affected households' evolving needs after two devastating earthquakes struck Nepal in April (M7.8) and May (M7.3) 2015 (The Asia Foundation, 2019). The IRM project team revisited the same disaster‐affected households and asked them similar questions over a 5‐year duration following the disaster (see Figure 2), collecting recovery evidence that goes beyond conventional one‐off postdisaster damage and needs assessments. Questions included, for instance, “to what extent was your livelihood affected by the earthquake?”, “do you or anyone else in your household plan to migrate in the next 12 months?” (which captures household relocation inclination), “how satisfied are you with the electricity?”, “approximately how much damage has the earthquake caused to your house?”, “how much of the NPR 300,000 grant (from the National Reconstruction Authority) have you received at this point?”, and “what is your household's source of income?”

In this study, we adopt the fourth‐round survey data (collected in April 2017; The Asia Foundation, 2017)—as opposed to previous survey rounds conducted during the emergency response (The Asia Foundation, 2015) and the early recovery phase (The Asia Foundation, 2016a, 2016b) when temporary displacement was the dominant migration pattern (The Asia Foundation, 2016b)—to focus on long‐term household relocation inclination. We do not adopt the fifth‐round survey data (collected between September and October 2019; The Asia Foundation, 2019) because any household relocation inclination observed at that point was not likely to be associated with the earthquakes in question given that “[t]he economy recovered in three years, 90% of people were back in their homes after four years, and …infrastructure and non‐domestic constructions took five years to rebuild and repair” (Platt et al., 2020).

The fourth‐round survey data include information on the respondent (e.g., age, income, gender, profession, broad ethnicity group, and educational attainment) as well as their household‐level characteristics (e.g., household size, annual household income). The survey data contain responses from 4854 households. Excluding households with “unknown” residential damage or “unknown” status of access to government funding leads to a total of 3300 complete responses (samples), which are used to develop the model. Among these responses, only 154 households are deemed to have had an inclination to relocate.

### 3.2 Selection of preliminary predictors

We first select a set of preliminary household‐level predictors to include in the **Data‐driven Model**, based on an extensive literature review of relocation following historical disruptive events (e.g., Badri et al., 2006; Comerio, 2014; Fussell et al., 2010; Ge et al., 2010; He et al., 2018; Henry, 2013; Myers et al., 2008; Peacock et al., 2014, etc.) including the 2015 Gorkha earthquakes, and broader studies on resilience and social vulnerability (e.g., Cutter et al., 2010, 2003, etc.).

The review identifies numerous factors influencing household relocation decision‐making (and therefore likely to be related to relocation inclination). These factors vary in prominence across different contexts (Henry, 2013; Paul et al., 2024), highlighting the importance of using bespoke models for characterizing household relocation decision‐making. Paul et al. (2024) grouped these factors into four broad categories: housing matters, financial aspects, social and community aspects, and demographics. Housing matters include housing (residential) damage, housing type (e.g., single‐family or multifamily), tenure time, or hometown status (which is also used by Nejat & Ghosh, 2016, as a proxy for place attachment). Financial aspects include property damage losses, whether the property is insured, and availability of external financial assistance such as government aids, grants, and loans (Alisjahbana et al., 2022). Social and community aspects include family and relationships, livelihood, neighborhood damage level, place satisfaction, and so forth. Demographics include housing tenure (i.e., renters or owners), income, age, gender, race and ethnicity, educational attainment, and so forth.

The eight initial (household‐level) predictors selected are residential damage, access to government funding (from the National Reconstruction Authority of Nepal), livelihood impact, place satisfaction, household income group, gender of the household head, age of the household head, and household size. Table 1 provides descriptions of these preliminary predictors and examples of literature that support their inclusion in the model.

**TABLE 1:** Preliminary household‐level predictors considered for inclusion in the case study **Data‐driven Model**. These predictors span the four categories of factors related to household relocation decision‐making identified by Paul et al. (2024).

Category	Predictor	Description	Literature
Housing matters	Residential damage	Whether the residence was damaged by the earthquakes. Possible categorical values: 0 (not damaged) and 1 (damaged).	Costa, Haukass, et al. (2022); Myers et al. (2008); Fussell et al. (2010); Peacock et al. (2014)
Financial aspects	Access to government funding	Whether the household has received a reconstruction grant from the National Reconstruction Authority of Nepal. Possible categorical values: 0 (not received) and 1 (received).	Comerio (2014); Alisjahbana et al. (2022); Kotani et al. (2020); Hamideh and Sen (2022)
Social and community aspects	Livelihood impact	Whether the livelihood of the household was impacted by the earthquakes. Possible categorical values: 0 (not impacted) and 1 (impacted).	Bolin and Bolton (1983); Wang et al. (2015); Henry (2013); Zhang and Peacock (2009); Cong et al. (2018); Comerio (2014); He et al. (2018)
	Place satisfaction	Whether the household is currently satisfied with its electricity and drinking water supply, schools, medical facilities, and roads. Possible categorical values: 0 (not satisfied) and 1 (satisfied).	Lu (1998); Tan (2016); Costa, Wang, et al. (2022a); Speare (1974)
Household demographics	Household income group	Possible categorical values: 1 (monthly income lower than 20,000 Rs, i.e., low income), 2 (monthly income between 20,000 and 40,000 Rs, i.e., middle income), and 3 (monthly income above 40,000 Rs, i.e., high income).	Ardayfio‐Schandorf (2012); Addo (2013, 2016); Cutter et al. (2003); Myers et al. (2008); Morrow‐Jones and Morrow‐Jones (1991)
	Gender of the household head	Possible categorical values: 1 (female) and 2 (male).	Cutter et al. (2003); Myers et al. (2008); Morrow‐Jones and Morrow‐Jones (1991)
	Age of the household head	Possible integer values: integers greater than 18.	Anton and Lawrence (2014); Nejat and Ghosh (2016); Clark et al. (2017); Speare (1974); Cutter et al. (2003)
	Household size	The number of individuals within the household. Possible integer values: positive integers.	Cutter et al. (2003); Durage et al. (2014); Xu et al. (2017)

Residential damage is often highlighted as an important factor in households' postdisaster relocation decision‐making (e.g., Costa, Haukass, et al., 2022; Fussell et al., 2010; Myers et al., 2008; Peacock et al., 2014). For instance, Myers et al. (2008) analyzed county‐level data from the US Census Bureau and found that disaster‐hit regions with more severe housing damage experienced greater out‐migration after Hurricanes Katrina and Rita.

Funding for housing repairs is essential to postdisaster recovery (Comerio, 2014). After a major disaster, government funds may be released to help affected households repair damaged (or build new) residences (Alisjahbana et al., 2022; Comerio, 2014; Hamideh & Sen, 2022; Kotani et al., 2020). In the absence of sufficient funding for housing repairs, housing abandonment and, therefore, household relocation (Zhang, 2012) frequently occurs. Relocation decision‐making is also tied to livelihood loss (e.g., Bolin & Bolton, 1983; Cong et al., 2018; He et al., 2018; Henry, 2013; Wang et al., 2015; Zhang & Peacock, 2009), which can be as important a factor as housing damage (Comerio, 2014).

Place satisfaction is the gratification felt when desires and aspirations for residence and neighborhood conditions are met (Lu, 1998; Tan, 2016). Households with low place satisfaction are more likely to relocate as they are more reluctant to take on debt and stay in temporary housing for prolonged periods to wait for housing repairs to finish (Costa, Wang, et al., 2022a).

Age of the household head plays a role in household relocation decision‐making because of its close association with place attachment. Older people often have higher place attachment than younger people because they have had more time to create deeper bonds with their neighborhood and region through various social ties (e.g., Anton & Lawrence, 2014; Clark et al., 2017; Nejat & Ghosh, 2016; Speare, 1974). People with high place attachment show reluctance to move away from places to which they are attached after a disaster (e.g., Anton & Lawrence, 2014; De Koning & Filatova, 2020; Johnson et al., 2020). For example, some households whose homes were destroyed by the 2015 Gorkha earthquakes stayed on their original land rather than relocating because of their emotional attachment to ancestral homes (The Asia Foundation, 2019).

Household income group, gender of the household head, and household size (as well as the age of the household head) relate to social vulnerability (Cutter et al., 2003). Morrow‐Jones and Morrow‐Jones (1991) analyzed national‐level disaster‐induced migration data and found that socially vulnerable groups, such as female‐headed households, are more likely to relocate after a disaster. Similar findings are provided by Myers et al. (2008) in the context of Hurricanes Katrina and Rita. In general, households in low‐income communities exhibit low or very little residential mobility (e.g., Ardayfio‐Schandorf, 2012; Addo, 2013, 2016). Durage et al. (2014) found that large households (consisting of more than two persons) are more concerned and aware of disasters and are more prompt in making positive decisions on disaster‐related mobility, for example, preemptive evacuation or postdisaster relocation.

A multitude of factors other than those included in our **Data‐driven Model** can influence household relocation decision‐making. However, the eight predictors we consider span all four categories of factors influencing household relocation decision‐making identified by Paul et al. (2024). Furthermore, they are often observed to be some of the most influential considerations related to postdisaster relocation decisions (see fig. 5 in Paul et al., 2024). They are therefore deemed to capture the complexity of household decision‐making as adequately as possible, especially considering the (computational) challenges associated with adding more predictors to a data‐driven model (e.g., overfitting, low interpretability; Hastie et al., 2009).

**FIGURE 3:**
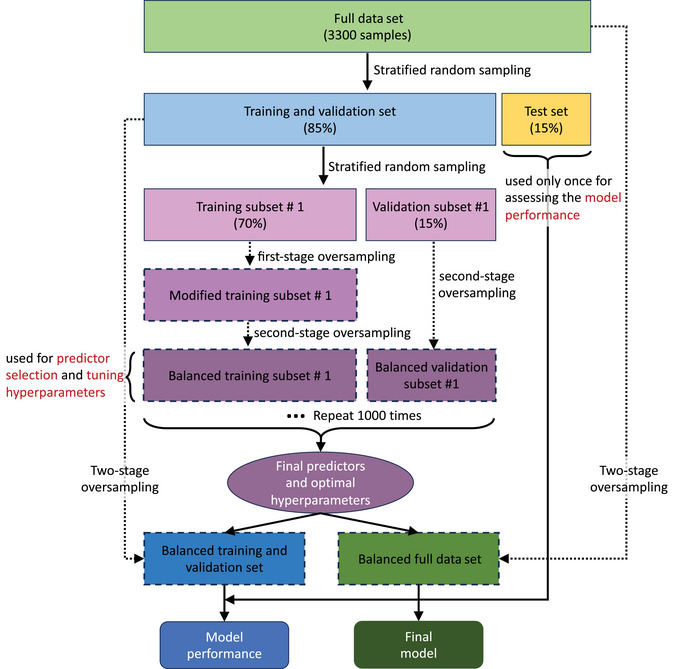
A brief snapshot of the modeling development process. The full data set is transformed into balanced training subsets, balanced validation subsets, and a test set. The balanced training and validation subsets are used to perform predictor selection and tune hyperparameters of the random forest model. The test data are reserved for assessing model performance.

**FIGURE 4:**
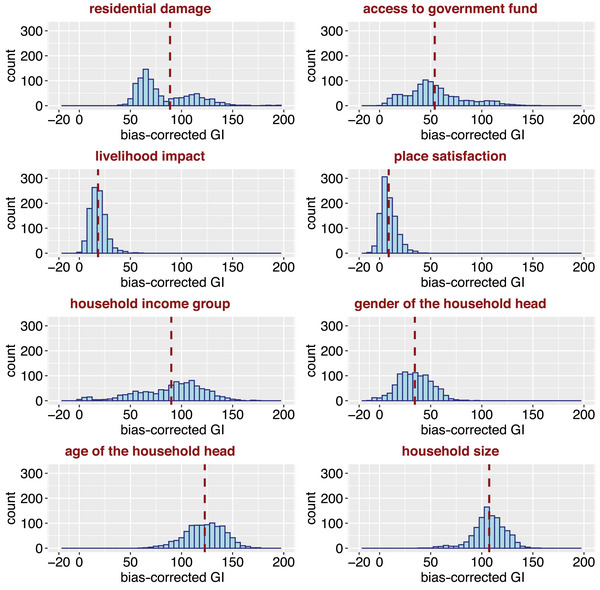
The distribution of bias‐corrected Gini importance (GI) of each predictor ($GI_{X_{i},r}$). The dark red dashed line indicates the mean bias‐corrected GI ($\hat{GI_{X_{i}}}$) of each predictor.

**FIGURE 5:**
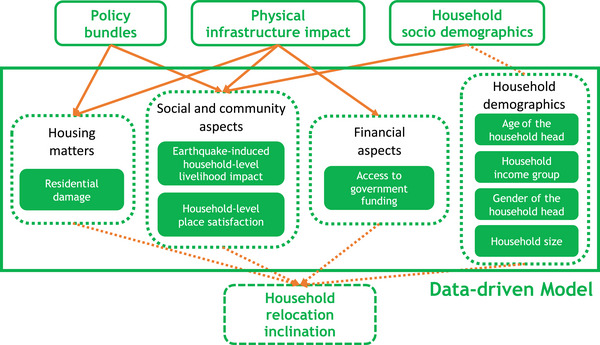
Schematic representation of the data‐driven model developed in this case study for assessing postearthquake relocation inclination of Nepali households. The data‐driven model holistically integrates various household‐level factors, that is, residential damage, livelihood impact, place satisfaction, access to government funding, age of the household head, household income group, gender of the household head, and household size.

### 3.3 Data preparation

The information required to characterize the predictors is then obtained from the IRM survey data. Positive relocation inclination is assigned to households that report at least one member currently planning to migrate in their responses to question D22. Residential damage is obtained from responses to question B1. Gender of the household head, age of the household head, and household size are obtained from responses to demographic questions (not numbered), assuming that the survey respondent is the household head. This assumption is justified, given that the survey respondent eligibility criteria stipulate that the respondent “plays an important role in the decision‐making process in the family.” We assume the earthquakes impacted the livelihoods of households who indicated that their jobs were “completely affected” or “somewhat affected” (for question C2). Data on access to government funding are obtained from responses to question F14. For place satisfaction, we assume households who indicated for question E2 that they were “somewhat dissatisfied” or “dissatisfied” with electricity, water, schools, medical facilities, or motorable roads are not satisfied. Household income group information is obtained by merging the income brackets reported by respondents for question A9 as follows: a low‐income household has a monthly income lower than 20,000 Rs, a middle‐income household has a monthly income between 20,000 Rs and 40,000 Rs, whereas a high‐income household has a monthly income above 40,000 Rs. These income groupings are determined based on the average monthly Nepali household income of 30,121 Rs (27,511 Rs for rural households and 32,336 Rs for urban households; Nepal in Data, 2018).

### 3.4 Model development

The **Data‐driven Model** is a random forest model (see Figure 1). We now present details on the development of this model, including the treatment of data, the structuring of the model, the selection of final predictors (Sandri & Zuccolotto, 2008), and model validation.

#### 3.4.1 Data treatment

The data are split into training subsets, validation subsets, and a test set, according to the procedure shown in Figure 3. A stratified random sampling technique (Reitermanova et al., 2010) is first used to split the full data set (3300 samples) into a combined training and validation set (85%), and a test set (15%), such that each set contains the same proportion of households with a positive inclination to relocate. We then randomly split the combined training and validation set into training (70%) and validation (15%) subsets 1000 times, such that each subset maintains the same proportion of households with positive relocation as the combined one.

#### 3.4.2 Oversampling

The majority (95.5%) of data samples are not associated with a positive inclination to relocate, which renders the data set imbalanced (He & Garcia, 2009). A model fit to (and validated using) imbalanced output data is biased toward the majority outcome. Moreover, a very small portion (2.6%) of data samples are associated with no residential damage, meaning this information may be overlooked in the model training phase. To mitigate these issues, we use oversampling (He & Garcia, 2009) to resample each of the training and validation subsets and obtain balanced subsets (Gao et al., 2021). We apply a two‐stage oversampling procedure to the training subsets. The first resampling stage involves oversampling from samples with no residential damage, which increases their presence within the training subsets by 20 times. The size of each modified training subset varies according to the number of samples with no residential damage in the original training subset. We then apply oversampling to the modified training subsets to obtain 1000 sets of balanced training subsets in which 50% of samples are associated with positive relocation inclination. We apply oversampling only once to the validation subsets to obtain balanced versions in which 50% of samples are associated with positive relocation inclination. (The first oversampling process is only required for a balanced model fit, and is therefore not applied to the validation subsets).

#### 3.4.3 Model structure

The **Data‐driven Model** is a random forest model (Breiman, 2001). This type of model is suitable for estimating postearthquake household relocation inclinations for two reasons: (1) it does not require any assumption to be made on the probability distributions of data; and (2) as a tree‐based method, it can naturally handle both categorical and continuous data (Breiman, 2001). The model's outcome is the probability of each household having positive inclination to relocate.

#### 3.4.4 Selection of final predictors

We pretune two hyperparameters of the random forest model (i.e., the number of trees to grow and the number of predictors randomly sampled as candidate predictors at each split), using a grid search on the 1000 balanced training and validation subsets. We then use the mean bias‐corrected Gini importance (GI) measure (Sandri & Zuccolotto, 2008) to identify predictors for the final model that consistently have predictive power (i.e., have a greater GI than random noise, which follows a uniform distribution between 0 and 1) across the 1000 training subsets, in line with Loos et al. (2023).

Mean bias‐corrected GI, $\hat{GI_{X_{i}}}$, is given by

(3) $$\hat{GI_{X_{i}}}=\frac{1}{R}\cdot \sum_{r=1}^{R}(GI_{X_{i},r}-GI_{Noise,r}),$$

where R=1000 is the number of models fit using random forest, $GI_{X_{i},r}$ is the GI associated with predictor $X_{i}$ in the rth model fit to the rth balanced training subset, and $GI_{Noise,r}$ is the GI associated with random noise in the rth model. A positive value of $\hat{GI_{X_{i}}}$ indicates that the associated predictor $X_{i}$ has a stronger predictive power compared to random noise, and the higher $\hat{GI_{X_{i}}}$ is, the more predictive power $X_{i}$ possesses.

Figure 4 provides the distribution of $GI_{X_{i},r}$ as well as $\hat{GI_{X_{i}}}$ for each candidate predictor. Residential damage, age of the household head, household size, and household income group have relatively higher $\hat{GI_{X_{i}}}$ values than the rest. All eight predictors have some predictor power in the random forest model (i.e., their associated $\hat{GI_{X_{i}}}$ values are positive), so are selected for inclusion in the final model.

GI is known to be biased in favor of predictors with many possible split points (e.g., categorical predictors with many possible categories, continuous predictors, and integer predictors; Nembrini et al., 2018), which feature in this case. Therefore, using a binary noise (which has much fewer possible split points than a uniform noise) could lead to a different set of predictors with positive $\hat{GI_{X_{i}}}$. Moreover, the performance of a statistical learning model can be significantly affected by its hyperparameters (Hastie et al., 2009). To understand the effects of the noise type and hyperparameters of the random forest model on the selection of final predictors, we repeat the final predictor selection process for random forest models fit using default (rather than pretuned) hyperparameters and a binary random noise (0 or 1). $\hat{GI_{X_{i}}}$ values associated with the untuned models are lower than those of Figure 4 (and some are close to zero), highlighting the importance of pretuning hyperparameters during the predictor selection process. We also find $GI_{Noise,r}$ values for binary noise to be lower than those obtained for uniform noise. The fact that all predictors would therefore have even larger $\hat{GI_{X_{i}}}$ tuned values in the presence of binary noise supports their inclusion in the final model.

#### 3.4.5 Tuning hyperparameters

We refit the model on the 1000 balanced training subsets using the eight predictors (excluding the noise) and tune the two hyperparameters by maximizing the average area under the curve (AUC) of the receiver operating characteristic (ROC) curve for the corresponding validation subsets (Huang & Ling, 2005). Once the optimal set of hyperparameters is identified, we refit the model on a balanced version of the combined training and validation set, which we obtain using the two‐stage oversampling technique described in Section 3.4.2.

#### 3.4.6 Model validation

**FIGURE 6:**
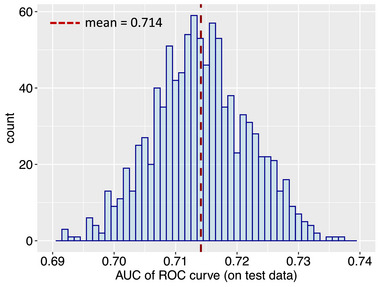
The distribution of the area under the curve (AUC) for the test data receiver operating characteristic (ROC) curves. The red dashed line represents the mean AUC value obtained (= 0.714).

We evaluate the performance of the refit model by calculating the AUC of ROC curves for the heldout test data. The test data represent hypothetical future data, so can be used to measure the refit model's “true” predictive power. Figure 6 shows the distribution of AUC values obtained for the test data across 1000 simulations in which slightly different random forest models are fit (due to randomness at each node split) using the same set of hyperparameters. The mean, maximum, and minimum AUC values obtained for the test set are 0.714, 0.741, and 0.677, respectively. Previous studies have considered an AUC value of 0.7 to indicate fair or moderate discriminative ability (e.g., Coroller et al., 2016; den Boer et al., 2005; Ferreira et al., 2022; Lee, 2014; Marcou & Rognan, 2007; Metz, 1986; Phillips et al., 2008; Suthar et al., 2013; Wang et al., 2022; Zhan & Chen, 2021). Therefore, we consider the performance of our model to be satisfactory, especially considering the limited number of samples with positive relocation inclination in the full data set. The final step involves using the two‐stage oversampling technique described in Section 3.4.2 on the full data set (see Figure 3) and refitting the **Data‐driven model** on the balanced version of this data set using the optimal set of hyperparameters.

## 4 CASE‐STUDY DESCRIPTION

We leverage the enhanced simulation‐based framework to investigate the effect of different disaster policies on mitigating postearthquake relocation inclination across households in the 11 districts most affected by the 2015 Gorkha earthquakes outside of the Kathmandu Valley, Nepal, using the **Data‐driven Model** developed in Section 3. We adopt the Tomorrowville expanding virtual urban testbed as our case‐study region (Menteşe et al., 2023), which was largely developed based on data from the Kathmandu Valley, recognizing the effectiveness of virtual testbeds as neutral spaces for testing community resilience analysis tools (Amin Enderami et al., 2022).

### 4.1 Urban Planning

**FIGURE 7:**
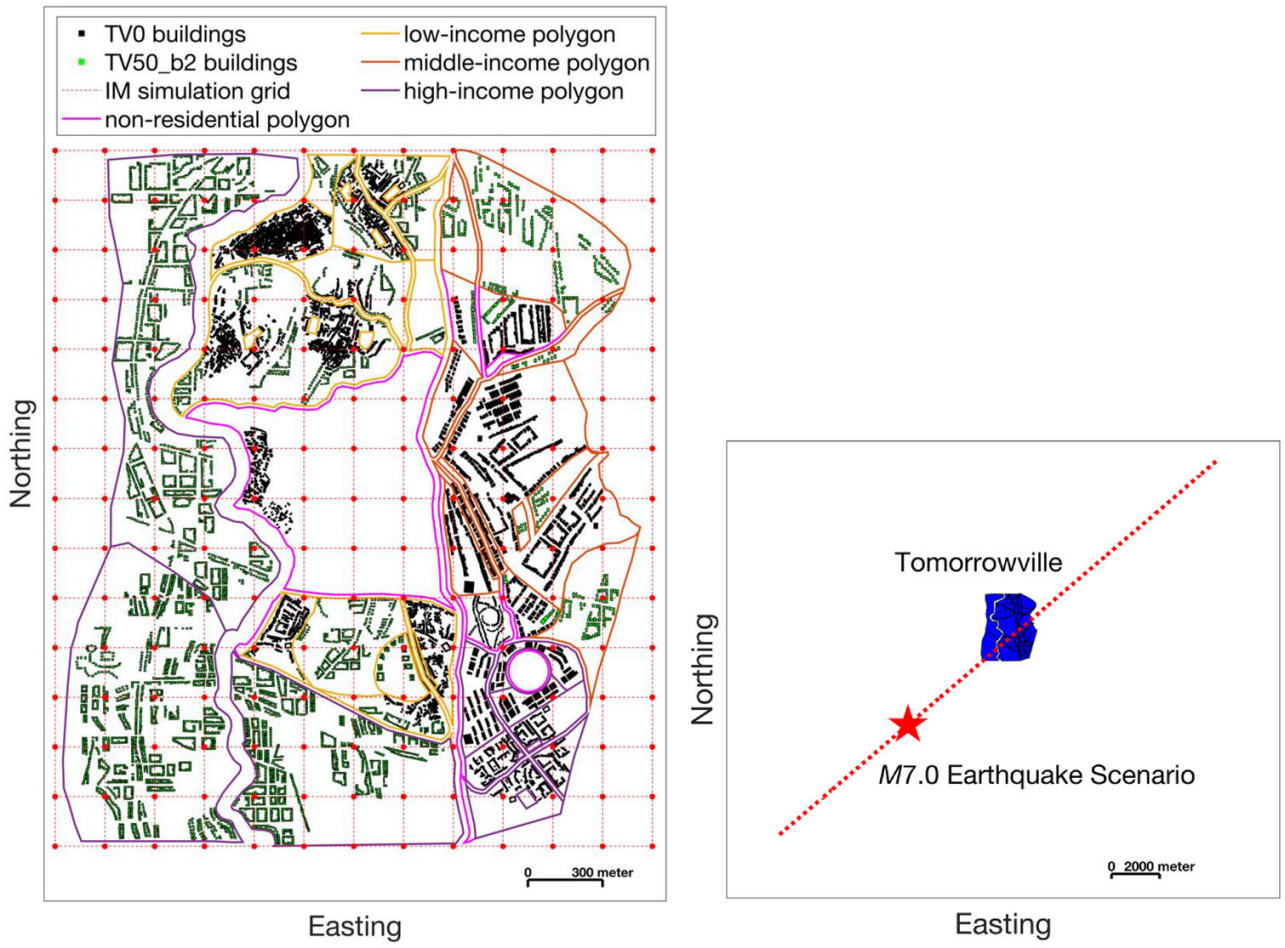
The left panel shows the buildings projected to be present in Tomorrowville in 50 years, as well as the associated land use polygons (TV50_total). TV50_total includes 8713 residential buildings and 1443 nonresidential buildings. Ground‐motion fields (GMFs) are simulated on a 200 m × 200 m grid (marked in red) across Tomorrowville polygons. The right panel shows the hypothetical M7.0 earthquake scenario considered for this case study. The underlying seismic source is a vertical strike‐slip fault that ruptures 24 km, as shown in red.

We use the TV50_total version of Tomorrowville, which includes 4810 existing buildings in today's Tomorrowville (TV0) and 5346 new buildings anticipated to be built in 50 years (TV50_b2) as a result of rapid urban expansion, shown in the left panel of Figure 7. TV50_total contains 8713 residential buildings and 1443 nonresidential (e.g., commercial, industrial, agricultural, and mix‐use) buildings. These buildings consist of 11 construction types; new buildings to be built in TV50_b2 are, on average, much stronger and more ductile than existing buildings in TV0 (see Gentile et al., 2022; Wang et al., 2023, for more details). There are three types of residential polygons (low‐, middle‐, and high‐income; see the left panel of Figure 7). Households within the same polygon all belong to the same income group. TV50_total includes 6766, 3059, and 7985 low‐income, middle‐income, and high‐income households, respectively. See Section 3.1 in Wang et al. (2023) for more details on TV50_total.

### 4.2 Policy Bundles

We consider four DRR policies for mitigating postearthquake household relocation inclination in TV50_total (see Table 2). Policy #1, which provides livelihood assistance funds to households in which at least one member is made unemployed by an earthquake event, is a “soft” (and compensatory) policy. We assume that this policy eradicates the effect of livelihood impact on household relocation inclination. The other policies, which involve upgrading the most vulnerable TV0 workplace and residential buildings to higher building codes, are “hard” (and corrective). Policy #4 is income‐based (i.e., targets only low‐income households) and is designed to explicitly facilitate pro‐poor outcomes. Policies #2, #3, and #4 demand intensive resources to improve the seismic vulnerability of 745, 2666, and 2248 buildings, respectively. Note that relevant buildings that act as both workplaces and residences are upgraded across policies #2 to #4.

**TABLE 2:** Policies considered for this case study. “RC” refers to reinforced concrete.

Policy	Type	Description
#1	Soft & compensatory	Provides livelihood assistance funds to households in which at least one member is made unemployed by an earthquake
#2	Hard & corrective	Replaces non‐RC workplace buildings (745 buildings in total) with high‐code RC buildings
#3	Hard & corrective	Replaces non‐RC residential buildings (2666 buildings in total) with high‐code RC buildings
#4	Hard & corrective	Replaces non‐RC low‐income residential buildings (2248 buildings in total) with high‐code RC buildings

### 4.3 Seismic Hazard

We consider a fictitious M7.0 earthquake scenario on a hypothetical vertical strike‐slip fault through Tomorrowville (shown on the right panel of Figure 7), given the synthetic nature of the case‐study testbed. We use the ground‐motion model in Campbell and Bozorgnia (2014) and the spatial and cross‐IM correlation model in Markhvida et al. (2018) to simulate spatial cross‐correlated GMFs across a 200 m × 200 m grid of Tomorrowville (as shown on the left panel of Figure 7). We use Monte Carlo sampling to simulate 500 sets of GMFs for different IMs required by the considered fragility models (see tab. 5 in Wang et al., 2023). Five hundred simulations are deemed appropriate, as this number produces stable social impact assessment results (see Section 5 for details). Ground‐motion IM values for each building are taken to be those simulated at the nearest grid point.

### 4.4 Physical Infrastructure Impact

We use fragility models associated with each building type to compute the DS of each building, conditional on the simulated IM values (outputs of the **Seismic Hazard** module). See Gentile et al. (2022) for details on the fragility models associated with Tomorrowville's buildings. The exact fragility models used are influenced by the three hard policies included in the **Policy Bundles** module.

The DS damage classification of the fragility models is translated to a binary residential damage classification to comply with the required input format of the **Data‐Driven Model**. DS=0 (“no damage”) is mapped to *Residential damage* =0, representing “not damaged.” DS=1 (“slight damage”), DS=2 (“moderate damage”), DS=3 (“extensive damage”), and DS=4 (“complete damage”) are mapped to *Residential damage* =1, representing “damaged” (FEMA, 2022).

### 4.5 Social Impact

The **Social Impact** module uses information from the **Physical Infrastructure Impact** module (i.e., the DS of each building and the converted residential damage classification), the **Urban Planning** module (e.g., the workplace buildings where employed individuals work, the age and gender of the household head, household income group, and household size), and the **Policy Bundles** module (i.e., how constituent policies affect the earthquake‐induced household‐level livelihood impact and residential building DS of each household) to quantify earthquake‐induced household‐level livelihood impact and the availability of government funding.

We assume that workplace buildings with at least extensive damage (DS≥3) cannot function, so the livelihoods of individuals working in these buildings are impacted. A household's livelihood is deemed to be impacted if the livelihoods of one or more of its employed members are impacted. We assume households with complete or extensive damage (DS≥3) to their residences will be provided with government funding. This assumption is consistent with the eligibility criteria for the reconstruction grant by the National Reconstruction Authority of Nepal after the 2015 Gorkha earthquakes (Amnesty International, 2017).

We randomly assign low place satisfaction to 40.7% high‐income, 39.9% middle‐income, and 34.6% low‐income households, in line with the respective proportions of each income group associated with low place satisfaction in the household survey data used (see Section 3.1 for details). Note that the relatively higher place satisfaction of low‐income households is consistent with observations in the literature. For example, Adriaanse (2007) found that low‐income households are usually associated with low residential mobility (e.g., Ardayfio‐Schandorf, 2012; Addo, 2013). They build up habitual routines over time and become psychologically fused with their residences, thereby having positive place satisfaction (Addo, 2016).

This module finally leverages the **Data‐driven Model** to compute the probability of having a positive relocation inclination for each TV50_total household across each GMF (i.e., Monte Carlo sample). We use a different random threshold value between 0 and 1 to translate this probability into a binary outcome ($I_{hh,j}=0$ or $I_{hh,j}=1$) for each Monte Carlo sample; $I_{hh,j}=1$ is assigned if the probability exceeds the threshold value and vice versa.

## 5 RESULTS

**FIGURE 8:**
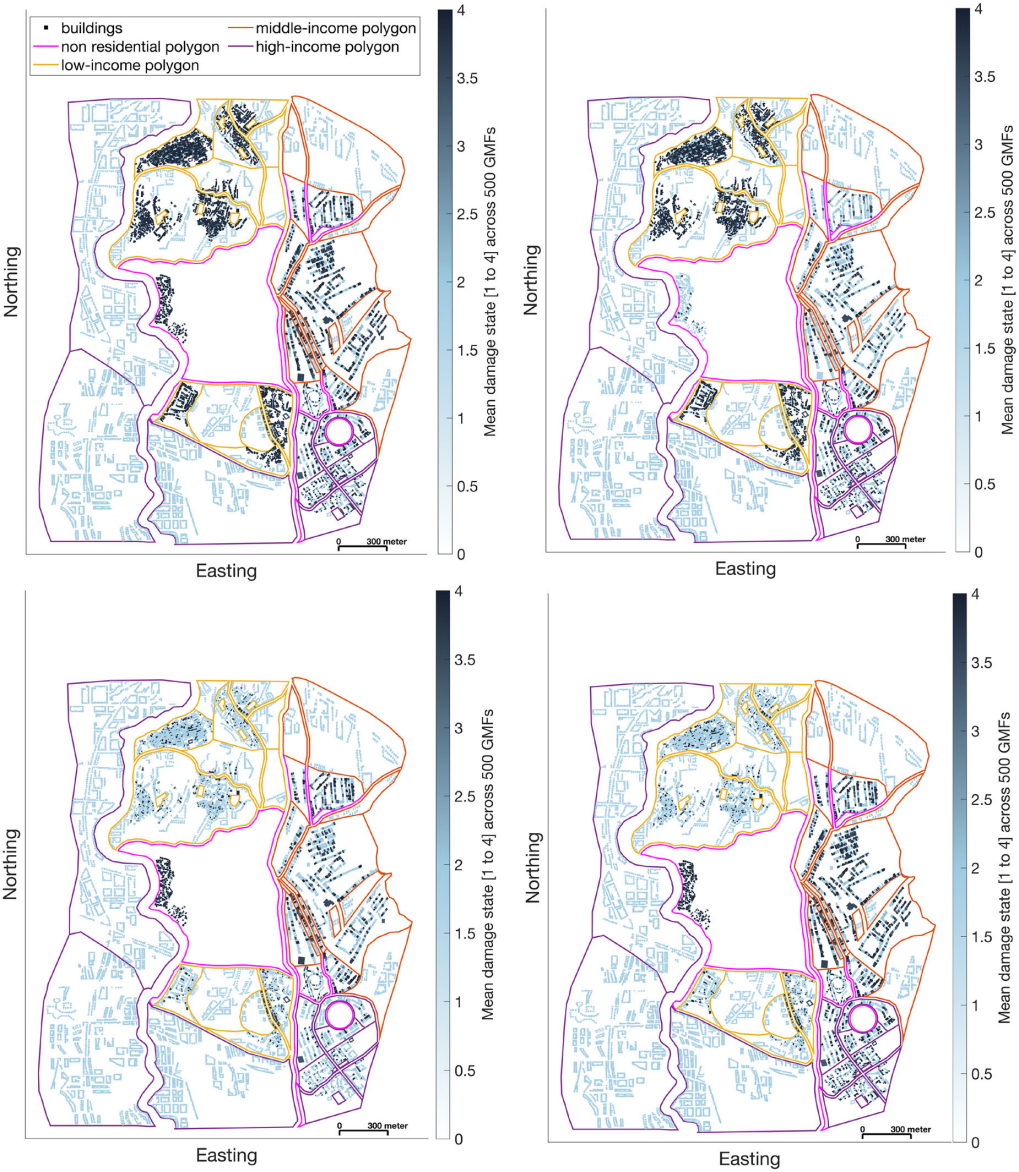
Average damage states DSs (0 = no damage, 1 = slight damage, 2 = moderate damage, 3 = extensive damage, and 4 = complete damage) of TV50_total buildings across the 500 sets of ground‐motion fields (GMFs) generated for the considered M7.0 earthquake scenario. The top left panel shows the results for the original TV50_total building portfolio, and the top right panel, bottom left panel, and bottom right panel show the results for policies #2, #3, and #4, respectively (see Section 4.2 for details).

Figure 8 displays the DSs of Tomorrowville buildings averaged across the 500 sets of GMFs generated for the considered M7.0 earthquake scenario and four building portfolios: the original TV50_total building portfolio (top left panel) and three upgraded building portfolios associated with policies #2 (top right panel), #3 (bottom left panel), and #4 (bottom right panel), respectively. The majority of buildings to be replaced under policy #3 are in the low‐income polygons. This explains why the policy noticeably reduces the positive difference between the average DSs of buildings in the low‐income polygons and those in the middle‐ and high‐income polygons. The results for policy #4 are a combination of the top left and the bottom left panels of Figure 8, that is, residential buildings in low‐income polygons are assigned the corresponding values shown in the bottom left panel, and all other buildings are assigned the corresponding values shown in the top left panel.

**FIGURE 9:**
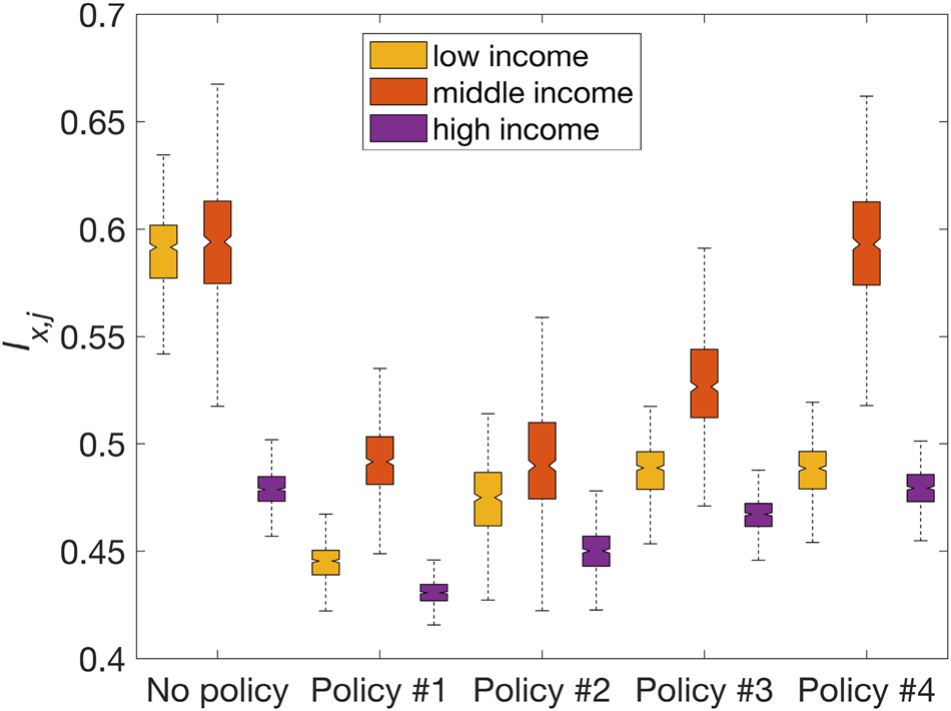
Box plots of $I_{x,j}$ for low‐, middle‐, and high‐income households under policies #1 to #4 (and no policy) resulting from the 500 sets of generated ground‐motion fields (GMFs).

Figure 9 displays box plots showing the proportions of low‐, middle‐, and high‐income households with positive relocation inclinations (i.e., $I_{x,j}$ in Equation 1) under policies #1 to #4 (and no policy), for each jth GMF. All four considered policies mitigate positive postearthquake relocation inclination of low‐income households (as expected). Policy #1 (soft and compensatory) is the most effective in mitigating positive postearthquake relocation inclination across all income groups. Hard policy #2 outperforms hard policy #3 in mitigating positive postearthquake relocation inclination. Moreover, policy #2 (which involves upgrading 745 buildings) requires much fewer engineering resources than policy #3 (which involves upgrading 2666 buildings) (see Section 3.2). Policy #4, which is a subset of policy#3, leads to the smallest reduction in the number of households with positive relocation inclination.

**FIGURE 10:**
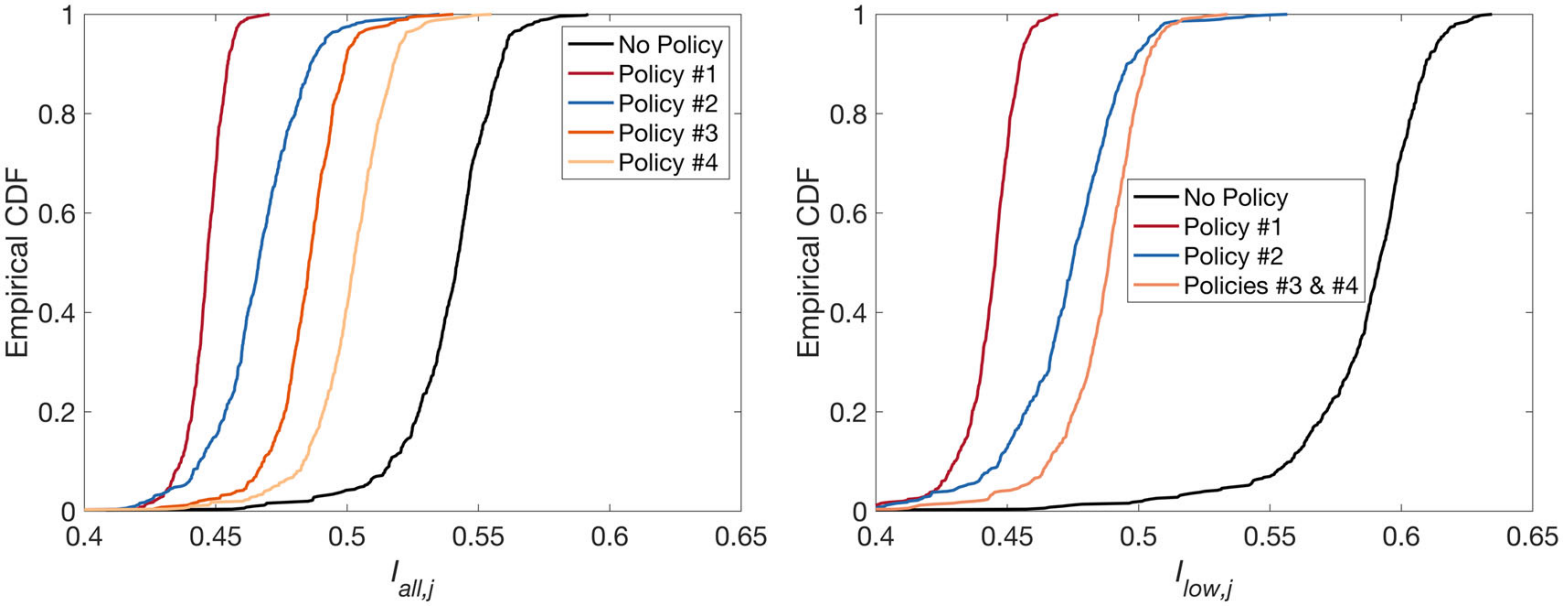
Empirical cumulative distribution functions (CDFs) of $I_{all,j}$ (left panel) and $I_{low,j}$ (right panel), for the 500 sets of ground‐motion fields (GMFs). The results are shown for policies #1 to #4 (and no policy).

Figure 10 shows for all policies (and no policy) the empirical cumulative distribution functions (CDFs) of $I_{all,j}$ (left panel) and $I_{low,j}$ (right panel), across the 500 sets of generated GMFs. Policies #1 and #2 are more effective than policies #3 and #4, both for all income groups and the low‐income one specifically. This highlights that in the context of Tomorrowville (and the underlying **Local Data**), earthquake‐induced household‐level impact on livelihood (related to policies #1 and #2) has a larger marginal impact on household postearthquake relocation inclination than the combined effects of government funding (determined based on residential DS) and residential damage (related to policies #3 and #4). Note that due to the binary classification of residential damage and the large extent of damage induced by the considered M7.0 earthquake scenario, there are only minimal changes in residential damage values across different policies. This means that policies #3 and #4 predominantly affect the access to government funding predictor only.

**FIGURE 11:**
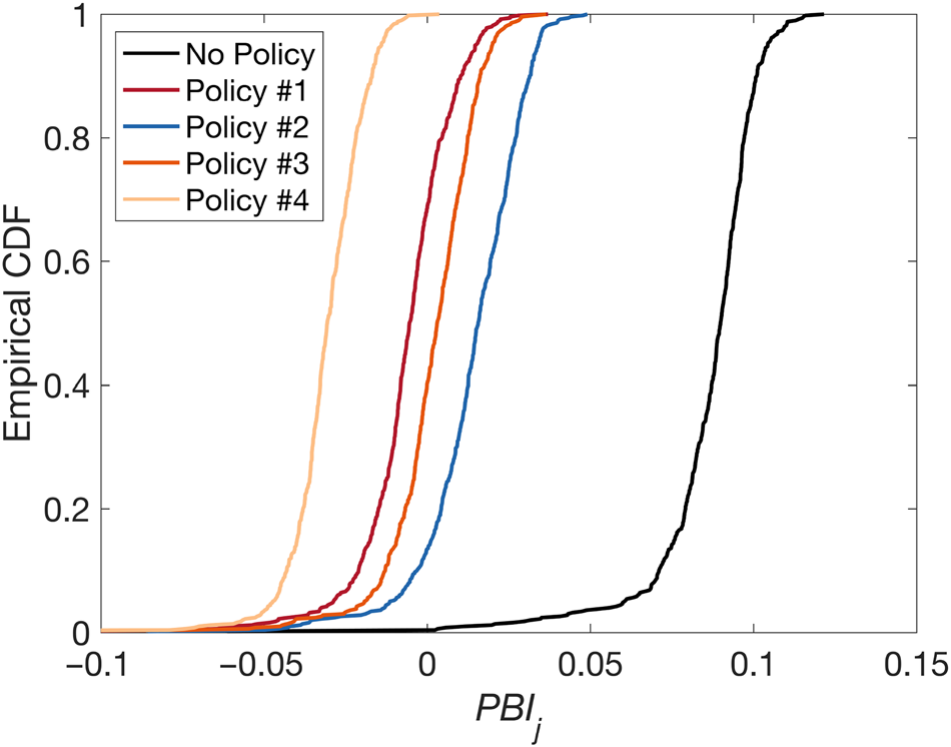
Empirical cumulative distribution functions (CDFs) of $PBI_{j}$ under policies #1 to #4 (and no policy) across the 500 sets of ground‐motion fields (GMFs).

Figure 11 shows for all policies (and no policy) the empirical CDFs of $PBI_{j}$ across the 500 sets of generated GMFs. All policies lead to some reduction in $PBI_{j}$. Policy #4 is consistently the most pro‐poor (i.e., it has the largest number of negative $PBI_{j}$ values) among those considered in this case study. This is expected given the low‐income remit of policy #4. Policy #1, a soft and compensatory policy that does not differentiate based on income, is associated with a negative $PBI_{j}$ for 343 GMFs (69% of Monte Carlo samples), making it the second most pro‐poor policy among those considered.

## 6 CONCLUSIONS AND FUTURE WORK

We present a new approach for assessing the effectiveness of DRR policies in mitigating positive postearthquake household relocation decision‐making. The approach involves enriching an existing framework that integrates social and physical considerations for risk‐informed policy design (Wang et al., 2023) with local data and an accompanying data‐driven model for estimating context‐specific postearthquake household relocation decision‐making.

We develop a random forest data‐driven model using Nepali household survey data collected in the wake of the 2015 M7.8 and M7.3 Gorkha earthquakes, to assess postearthquake relocation inclinations of local households. This model accounts for various household‐level factors related to postearthquake household relocation decision‐making, that is, residential damage, access to government funding, livelihood impact, place satisfaction, household income group, gender of the household head, age of the household head, and household size. In light of the general lack of (data‐driven) models focusing on household relocation inclination (or decision‐making), the model developed here serves as a novel risk‐sensitive planning tool that provokes discussion on a complex multidisciplinary social phenomenon.

We demonstrate the enhanced framework and the data‐driven model developed by assessing the effects of multiple DRR policies for an expanding virtual urban testbed Tomorrowville, which is largely informed by data from the Kathmandu Valley, Nepal. We particularly focus on the extent to which the policies mitigate positive postearthquake relocation inclination among low‐income households. The case study reveals that a soft policy of postdisaster livelihood assistance provision for all households impacted by earthquake‐induced unemployment (policy #1) is more effective in mitigating positive postearthquake relocation inclination than hard policies centered on the seismic strengthening of physical infrastructure (policies #2, #3, and #4). This emphasizes the fact that hard strategies, consisting of resource‐intensive engineering interventions, might not always be the most effective seismic risk reduction solution for urban areas exposed to seismic hazard. We also find that policy #1 is pro‐poor overall (i.e., has a negative mean $PBI_{j}$ value), despite providing assistance to households of all income groups. While this finding is limited to the case study's specific context, it suggests that opportunities exist for designing pro‐poor DRR policies without the need to explicitly account for income thresholds, which can be politically sensitive (Lyon & Sepulveda, 2009).

Our framework is explicitly forward‐looking, that is, it quantifies earthquake risks of urban communities accounting for uncertain future development in yet‐to‐be urbanized regions. Many of these regions (e.g., the Kathmandu Valley, Nepal) are experiencing rapid expansion and population growth, which could significantly intensify natural‐hazard exposure and vulnerability in the absence of risk‐sensitive planning tools and policies like those proposed here (Mesta et al., 2022, 2023). A forward‐looking perspective is particularly important for designing DRR policies related to postearthquake household relocation decision‐making; our framework can help to prevent relocation‐related accumulation of vulnerabilities from the outset and address the root causes of exacerbating inequalities in the wake of a future earthquake disaster.

While we focus on postearthquake household relocation in this study, our framework is sufficiently flexible to be extended to account for other context‐specific social impacts of earthquake disasters. For example, we could leverage the framework to provide a holistic characterization of postearthquake business interruption. In this case, data on how local business people make recovery decisions after a historic earthquake event could be used to develop a data‐driven model for characterizing business interruption. The model may depend on building and transportation network downtime, as well as resilience tactics that businesses can employ to hasten recovery (Cremen et al., 2020), for instance.

## CONFLICT OF INTEREST STATEMENT

The authors declare no conflicts of interest.

## Data Availability

The data and code for implementing the case study on Tomorrowville are made available through the following repository: https://github.com/wangcb98/household-relocation. Original data related to Tomorrowville can be found here: https://github.com/TomorrowsCities/Tomorrowville. The Nepali household recovery survey data can be requested from The Asia Foundation Nepal Country Office at nepal.general@asiafoundation.org.
